# Retrospective validation of 3D Slicer against CTA source images for accurate intracerebral hematoma volumetry: a comparative study with the Tada formula

**DOI:** 10.1186/s12880-025-02074-5

**Published:** 2025-11-22

**Authors:** Minghui Lu, Zohaib Shafiq, Wei Yang, Pei Liu, Shimin Tan, Jiajun Wei, Qiang Cai

**Affiliations:** 1 Department of Neurosurgery, Renmin Hospital of Wuhan University, No. 238, Jiefang Road, Wuchang District, Wuhan City, Hubei Province 430060 China; 2 Radiology Department, Xiantao First People’s Hospital, No. 29, Middle Section of Mianzhou Avenue, Nancheng New District, Xiantao City, Hubei Province 433000 China

**Keywords:** Intracerebral hemorrhage, Hematoma volume, 3D Slicer software, CTA source images, Tada formula, Accurate calculation

## Abstract

**Background:**

Accurate measurement of intracerebral hemorrhage (ICH) volume is critical for clinical decisions and prognosis. While the Tada formula (ABC/2) is commonly used, its accuracy—particularly for irregular hematomas—remains limited. This study retrospectively validates the free, open-source software 3D Slicer against manual segmentation of CTA source images as the gold standard, and compares its performance with the ABC/2 method.

**Methods:**

This study retrospectively analyzed imaging data from 118 patients with spontaneous ICH who underwent both non-contrast CT (NCCT) and CTA. The CTA images were acquired with thin-slice reconstruction (0.625 mm slice thickness). Hematoma volume was measured by two blinded evaluators using the following methods: (1) manual segmentation on thin-slice CTA source images (gold standard); (2) semi-automatic segmentation on NCCT images using 3D Slicer software; and (3) calculation on NCCT images using the Tada formula (ABC/2). Intraclass correlation coefficient (ICC) was used to assess intra- and inter-observer agreement. Bland-Altman analysis and linear regression were employed to evaluate the agreement and bias between 3D Slicer, the ABC/2 method, and the gold standard.

**Results:**

Analysis of 118 spontaneous intracerebral hemorrhage patients revealed that the 3D Slicer method achieved near-perfect agreement (ICC = 0.997) with the gold standard of CTA manual segmentation, with a minimal mean bias of + 0.77 mL and narrow limits of agreement. In contrast, the Tada formula demonstrated significantly poorer agreement (ICC = 0.887), a substantial systematic overestimation (mean bias = + 4.65 mL), and wide limits of agreement (-10.8 to + 20.2 mL). Subgroup analyses further confirmed the superior robustness of 3D Slicer. The reliability of the Tada formula was markedly lower for irregularly shaped hematomas (ICC = 0.835) and deteriorated drastically for large hematomas > 60 mL (ICC = 0.516).

**Conclusion:**

In conclusion, this study validates 3D Slicer as a highly accurate tool for ICH volumetry using CTA source images, demonstrating superior performance over the Tada formula, especially for irregular hematomas. As a freely available platform, it represents a superior alternative for precise ICH assessment in both clinical and research settings.

## Introduction

Spontaneous intracerebral hemorrhage (ICH) is a disease with high mortality and disability rates [1], and is one of the leading causes of long-term neurological deficits worldwide [2]. In the acute management of ICH, hematoma volume is one of the most critical imaging indicators for assessing disease severity, predicting patient outcomes, and guiding clinical decision-making, such as surgical intervention [3]. Therefore, the development of a fast, accurate, and reliable volume measurement method remains a major research focus in the fields of neurosurgery and neurocritical care.

Traditionally, the Tada formula (ABC/2) has been the default standard for ICH volume measurement in global clinical practice due to its unparalleled simplicity [4]. However, its geometric assumption of simplifying the complex three-dimensional hematoma morphology into an ellipsoid is an inherent and fundamental limitation of this formula [5]. Extensive literature has shown that the Tada formula exhibits significant and unpredictable measurement errors when applied to irregularly shaped, lobulated, or other complex hematomas, often resulting in either overestimation or underestimation of the hematoma volume [6–8]. Such inaccuracies may lead to entirely different treatment strategies when the hematoma volume approaches critical clinical decision thresholds, such as 30 mL [9].

With the advancement of medical imaging, volumetric analysis based on manual or semi-automatic segmentation has been widely recognized as a more accurate alternative for intracerebral hemorrhage (ICH) assessment [10]. Among various software tools, 3D Slicer (version 4.11, http://www.slicer.org) [11], a free, open-source, and powerful platform, has demonstrated excellent performance in research settings for volume quantification [12]. However, previous validation studies of 3D Slicer have been limited by a methodological concern: the common practice of using manual segmentation on non-contrast CT (NCCT) images as the reference “gold standard.” This approach may introduce bias, as the segmentation accuracy is evaluated against the same modality it derives from, rather than an independent standard.

In this study, we utilize source images from computed tomography angiography (CTA), to perform a comparative assessment of the ABC/2 method and 3D Slicer segmentation. Under the imaging protocol employed in this study, the utilized CTA source images were characterized by submillimeter slice thickness (0.625 mm slice thickness), isotropic voxels, and high spatial resolution. These technical attributes facilitated the delineation of brain parenchyma, hematoma boundaries, and normal vascular structures, as detailed in the Methods section [13]. The rationale for selecting thin-slice CTA as the reference standard was based, in part, on its ability to mitigate partial volume effects through high-resolution imaging. This approach provides a more robust methodological foundation for the segmentation accuracy assessment [14]. Therefore, the manually segmented volume based on CTA source images serves as a feasible and authoritative highest standard in this study.

The purpose of this study is to retrospectively evaluate the accuracy of 3D Slicer for measuring intracerebral hemorrhage (ICH) volume by using manual segmentation on computed tomography angiography (CTA) source images as the gold standard. A rigorous comparison was conducted between 3D Slicer and the Tada formula to precisely quantify the magnitude and patterns of measurement errors associated with the latter in real-world clinical data. Furthermore, subgroup analyses were performed to thoroughly investigate the influence of hematoma morphology and size on the accuracy of both measurement methods.

## Methods

### Patient population

This single-center, retrospective, observational study utilized the electronic medical record system of Renmin Hospital of Wuhan University to consecutively retrieve imaging records of all patients admitted with a primary diagnosis of acute spontaneous intracerebral hemorrhage (ICH) between January and December 2023.

Inclusion criteria were as follows: (1) age ≥ 18 years; (2) initial CT confirmation of supratentorial spontaneous ICH including cases with intraventricular extension; (3) brain CT and CTA were sequentially acquired during the same initial imaging session within 24 h of admission. Exclusion criteria included: (1) secondary ICH (e.g., rupture of aneurysm, arteriovenous malformation, moyamoya disease, tumor stroke, or trauma); (2) history of major brain surgery or large-area cerebral infarction leading to structural deformation; (3) severe motion or metal artifacts on CT/CTA affecting accurate measurement; (4) missing CTA source image data.

**Fig. 1:**
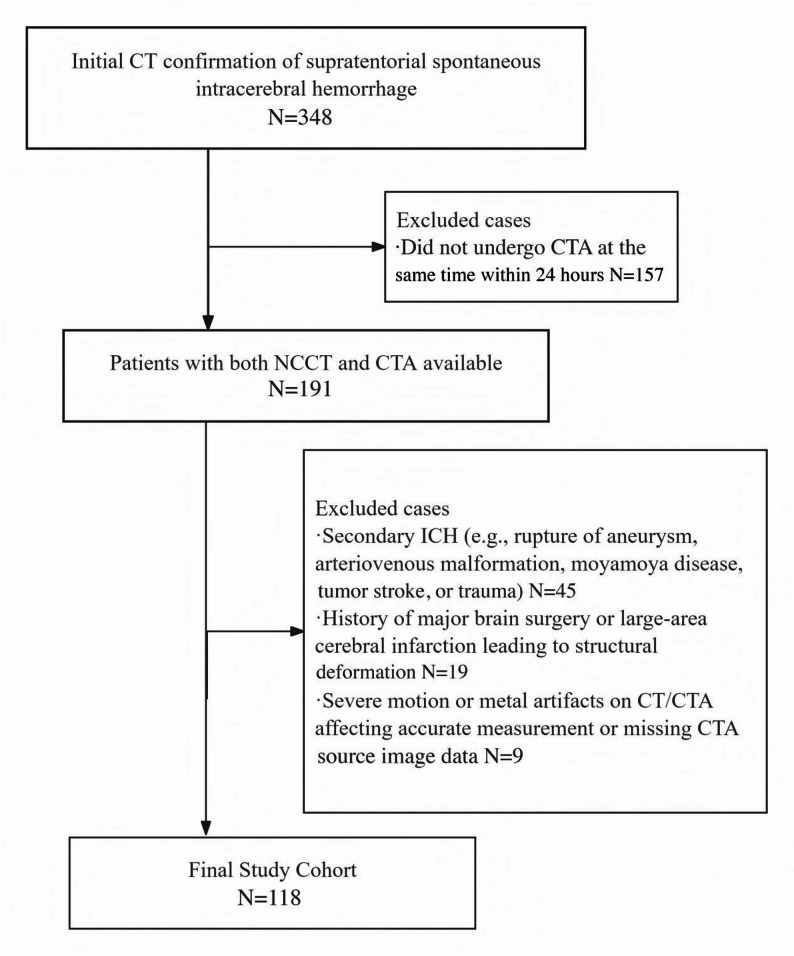
Flow chart for patient selection. The flowchart details the two-stage patient selection process. All 191 included patients underwent NCCT and CTA concurrently. The final cohort of 118 patients was derived after applying all exclusion criteria to this initial group

A total of 191 patients received both NCCT and CTA source images concurrently during the same initial imaging session. From this cohort, 73 patients were subsequently excluded based on specific clinical and radiological criteria resulting in a final analysis population of 118 patients. The patient screening process is detailed in Fig. 1.

Ethical approval for this study was obtained from the ethics committee at Renmin Hospital of Wuhan University. The requirement for informed consent was waived due to the use of fully anonymized data and the retrospective nature of the study, which was based solely on the analysis of existing medical images.

### Data collection

Brain CT and CTA scans from the final study cohort were retrieved from the hospital’s Picture Archiving and Communication System (PACS) in DICOM format. All non-contrast CT scans were acquired with a 5-mm slice thickness in contiguous mode. CTA was performed using a bolus-tracking technique after intravenous contrast injection. Thin-slice (0.625 mm) axial source images were reconstructed from raw data and used for subsequent analysis. All imaging was conducted on a Siemens Somatom Force scanner following a standardized institutional acute stroke protocol. The native scans in our institutional protocol did not include source files with 0.6 mm thickness.

Regular hematoma is a hematoma that is relatively regular in shape on CT or MRI images, mostly in the form of a round-like, elliptical, or homogeneous mass, with well-defined borders, smooth edges, and no obvious lobes or protrusions. Irregular hematomas are irregular in shape, with blurred or lobulated or multilocular margins, and may be accompanied by “satellite foci” (small peripheral hemorrhages) with uneven density/signal. Two experienced neurosurgeons work together to distinguish whether a hematoma is regular or not.

### Hematoma volume measurement

All image analyses were performed by two independent evaluators (both were attending neuroradiologists with over 5 years of experience), who were blinded to each other’s results and clinical data. The order of measurements was randomized.

#### Gold standard

Manual Segmentation on CTA Source Images: CTA source images (0.625 mm slice thickness) in DICOM format were imported into 3D Slicer (v5.2.2). Using the “Segmentation” module, the evaluator manually delineated the hematoma region of interest (ROI) slice by slice with the “Paint” tool. The segmentation process involved automatic detection of pixels within a predefined Hounsfield unit (HU) range (50–100 HU), followed by manual refinement using the scissors and erase tools to ensure precise outlining. This procedure was performed in all three anatomical planes: axial, coronal, and sagittal. A 3D model of the hematoma was reconstructed and thoroughly reviewed and modified to ensure anatomical accuracy. The “spot sign” was not evaluated in this study. All segmentation procedures were performed with concurrent reference to native non-contrast CT (NCCT) images to ensure accurate differentiation between ICH and other structures such as large vessels, tumors, or aneurysms. Throughout the process, the high resolution of CTA images facilitated clear discrimination between hyperdense acute hematoma and enhanced normal vascular structures. The software automatically computed the total hematoma volume (mL). Two independent evaluators performed the segmentation once each, and the average of their results was used as the final gold standard volume(GSV).

#### Calculation of hematoma volume using 3D Slicer

Non-contrast CT (NCCT) images with a slice thickness of 5 mm were imported into 3D Slicer. An initial segmentation was performed using the “Threshold” tool to select voxels within a Hounsfield unit (HU) range of 45–100, roughly encompassing the hematoma region. This was followed by meticulous manual refinement by the evaluator using the “Paint” and “Erase” tools to ensure that the segmentation boundaries accurately matched the visually identified margins of the hematoma. The procedure was conducted in all three anatomical planes—axial, coronal, and sagittal. A three-dimensional model of the hematoma was then reconstructed, thoroughly inspected, and optimized to ensure anatomical accuracy. The total volume was automatically computed by the software. Two independent evaluators performed the entire process once each, and the average of their results was adopted as the final volume(3DS-V).

#### Calculation of hematoma volume using the Tada formula

**Fig. 2:**
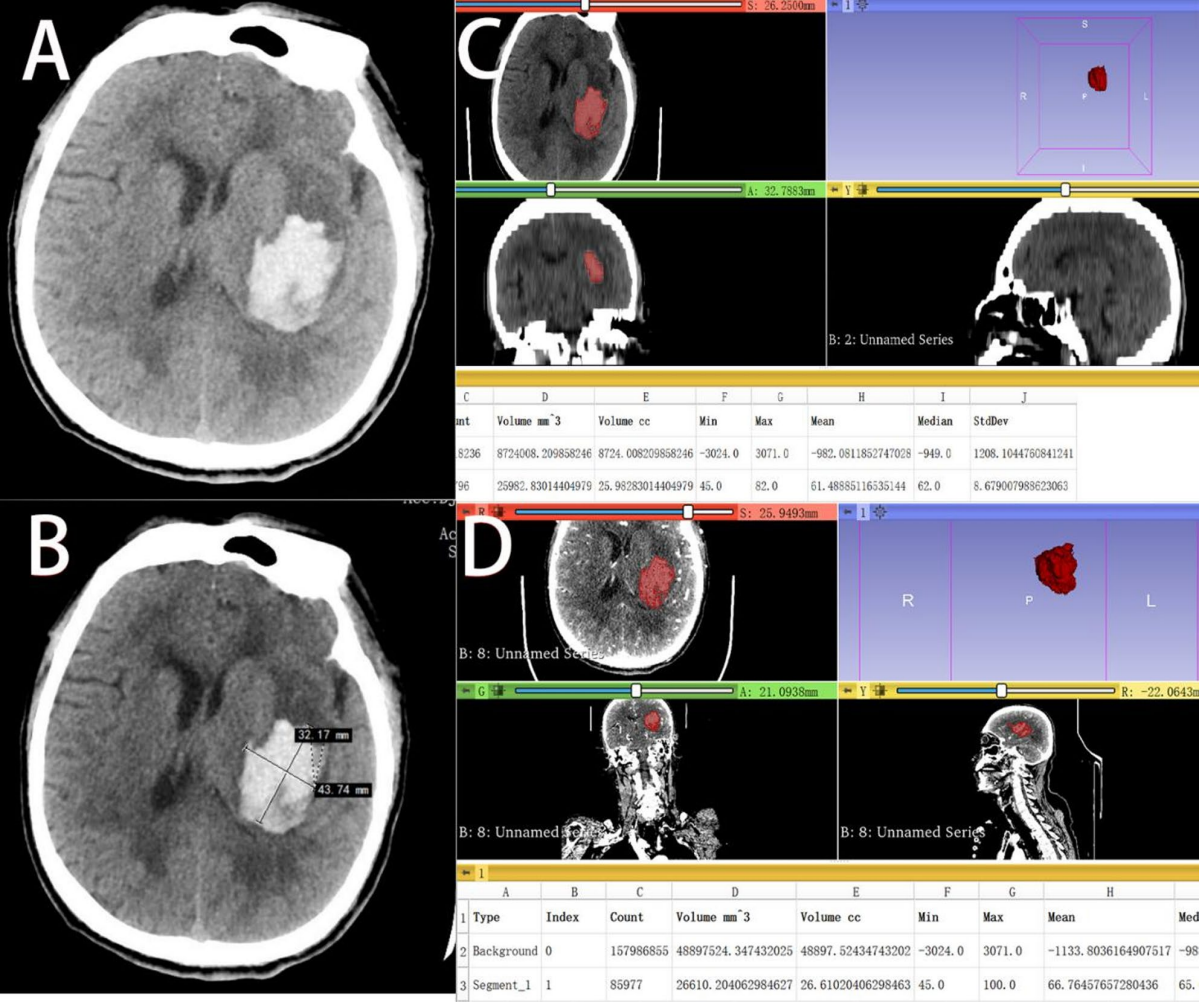
Comparison of Regular Hematoma Volume Calculations Using 3D Slicer and the Tada Formula. (**A**) Preoperative CT scan of the patient, showing the initial hematoma. (**B**) Preoperative hematoma volume calculated using the Tada formula (Tada-V), resulting in a volume of 24.6 mL. (**C**) Preoperative hematoma volume measured using 3D Slicer(3DS-V), resulting in a volume of 26.0 mL. (**D**) Preoperative hematoma volume was manually segmented on CTA source images(GSV), showing a volume of 26.6 mL

**Fig. 3:**
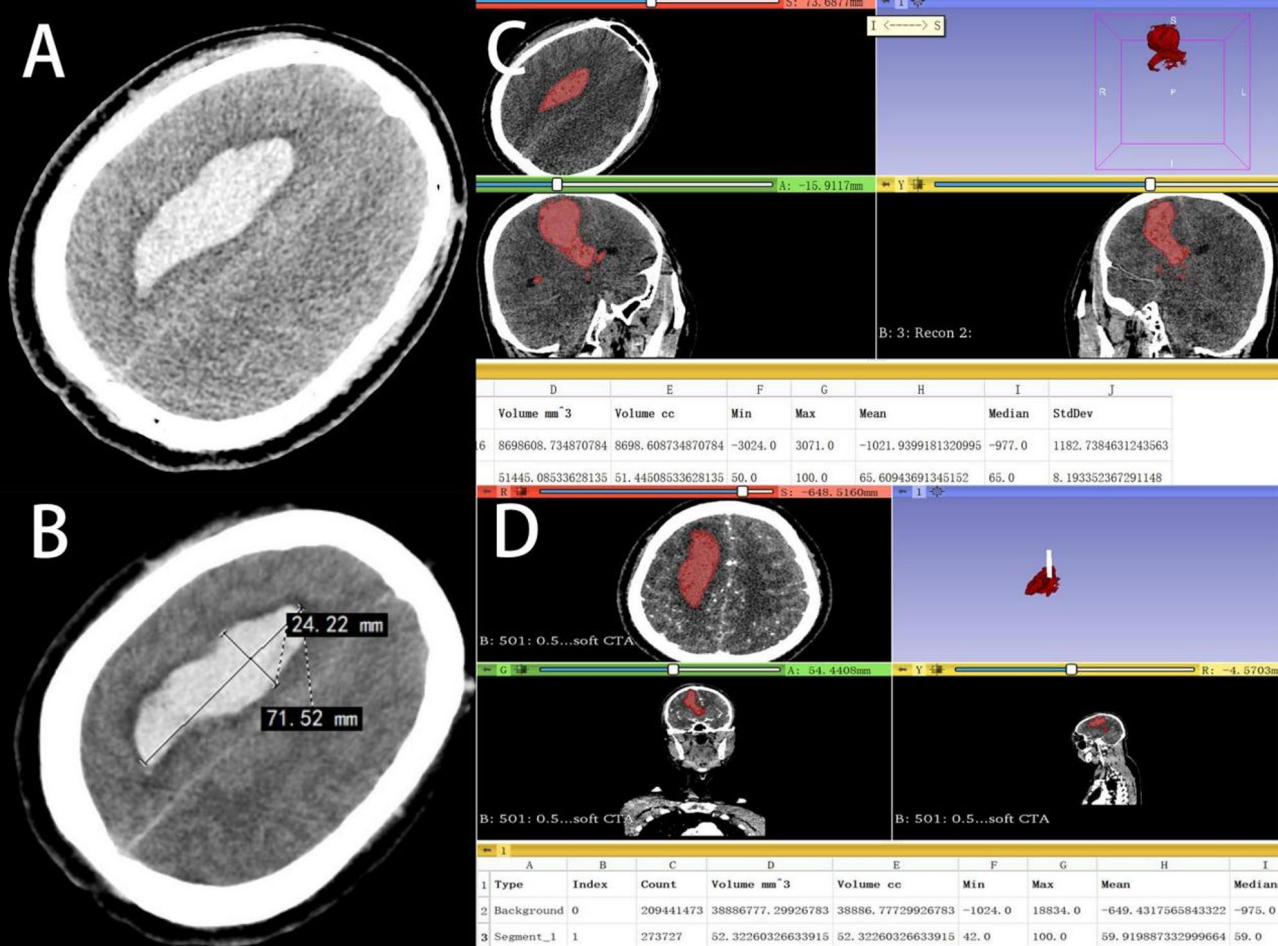
Comparison of Hematoma Volume Calculations for Irregular Hematomas Using 3D Slicer and the Tada Formula. (**A**) Preoperative CT scan of the patient, showing the initial hematoma. (**B**) Preoperative hematoma volume calculated using the Tada formula (Tada-V), resulting in a volume of 65.0 mL. (**C**) Preoperative hematoma volume measured using 3D Slicer(3DS-V), resulting in a volume of 51.4 mL. (**D**) Preoperative hematoma volume was manually segmented on CTA source images(GSV), showing a volume of 52.3 mL

On NCCT images, the evaluator identified the slice with the largest hematoma cross-section and measured the longest diameter (A) and the longest perpendicular diameter (B) within that slice. The value C was calculated as the number of slices containing the hematoma multiplied by the slice thickness (5 mm). The hematoma volume was then computed using the formula: ABC/2. This process was performed independently by two evaluators, and the average of their measurements was adopted as the final volume(Tada-V)(Figs. 2 and 3).

### Statistical analysis

Continuous variables that followed a normal distribution were reported as means with standard deviations, whereas those that did not adhere to a normal distribution were presented as medians along with their interquartile ranges (lower quartile to upper quartile). Consistency was evaluated using the intraclass correlation coefficient (ICC) along with its 95% confidence interval (CI). An ICC value greater than 0.90 was considered indicative of excellent agreement. Bland–Altman analysis was applied to calculate the mean bias and 95% limits of agreement (LoA). Subgroup analyses were conducted based on hematoma morphology and volume to compare the errors between the two measurement methods. Linear regression was employed to analyze the correlation between the absolute error of the Tada formula (|Tada-V − GSV|) and hematoma volume as well as morphology. Statistical analyses were conducted using SPSS version 24.0. A p-value of less than 0.05 was deemed statistically significant.

## Results

### Patient baseline characteristics

A total of 118 patients with spontaneous intracerebral hemorrhage (ICH) were included in the final analysis. The baseline and clinical characteristics of the cohort are summarized in Table 1. The study population had a mean age of 58.0 ± 13.3 years and was predominantly male (73.7%). The distribution of hematoma locations, with secondary intraventricular extension categorized under the primary site of hemorrhage, was as follows: basal ganglia (61.9%), followed by lobar (20.3%) and thalamic (17.8%) regions. The majority of hematomas (60.2%) were classified as irregular in shape. The remaining 47 hematomas (39.8%) were classified as regular.

**Table 1:** Baseline characteristics of the study cohort (*N* = 118)

Characteristics	Measurements
**Gender,** *** n*** ** (%)**	
male	87 (73.7%)
female	31 (26.3%)
**Age**,** mean ± sd**	58.0 ± 13.3
**Groups divided by shape**,** n (%)**	
Regular	47 (39.8%)
Irregular	71 (60.2%)
**Location of hematoma**,** n (%)**	
Basal Ganglia	73 (61.9%)
Thalamus	21 (17.8%)
Lobar	24 (20.3%)

### Volumetric measurements and method comparison

**Fig. 4:**
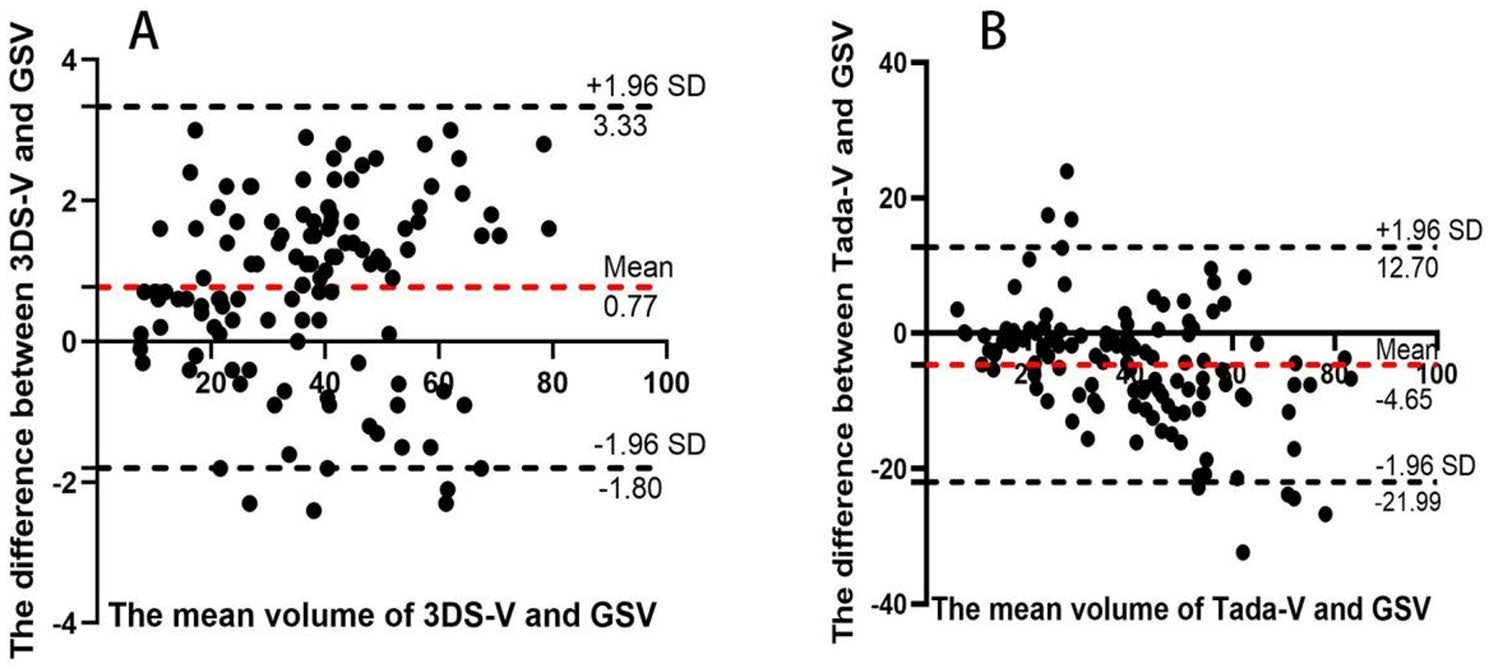
Bland-Altman plots were constructed to compare (**A**) 3D Slicer and (**B**) the Tada formula against the gold standard volume. The red dashed line represents the mean bias, and the dotted lines indicate the 95% limits of agreement. Note the significant positive bias and wide limits of agreement for the Tada formula

A summary of hematoma volumes quantified using the three different methods is provided in Table 2. Manual segmentation on CTA source images (GSV) yielded a mean hematoma volume of 37.92 ± 16.70 mL. The median volume was 38.55 mL (IQR: 24.13–48.50 mL), with an overall range from 7.5 to 80.1 mL, confirming the inclusion of a wide spectrum of hematoma sizes in the cohort. The 3D Slicer method (3DS-V) demonstrated excellent agreement with the gold standard, with a negligible mean bias of + 0.77 mL and an intraclass correlation coefficient (ICC) of 0.997 (95% CI: 0.996–0.998). In contrast, the Tada formula (Tada-V) exhibited systematic overestimation of hematoma volume, with a mean positive bias of + 4.65 mL relative to the gold standard and wide 95% limits of agreement (LoA) ranging from − 10.8 mL to + 20.2 mL, indicating substantial and unpredictable measurement errors. Furthermore, agreement between the Tada formula and the gold standard was significantly poorer, as evidenced by an ICC of 0.887 (95% CI: 0.841–0.920)(Fig. 4).

**Table 2:** Comparison of hematoma volumes measured by three different methods (mL)

Measurement method	Mean ± sd	Median (IQR)	Range	Bias (vs.GSV)	ICC(95% CI) (vs.GSV)
CTA Source Images (GSV)	37.92 ± 16.70	38.55 (24.13, 48.50)	7.5–80.1		
3D Slicer (3DS-V)	37.15 ± 16.61	37.30 (23.75, 48.20)	7.6–78.5	0.77	0.997 (0.996–0.998)
Tada formula (Tada-V)	42.57 ± 20.32	42.25 (24.38, 55.50)	4.3–91.5	-4.65	0.887 (0.841–0.920)

### Overall agreement trends

**Fig. 5:**
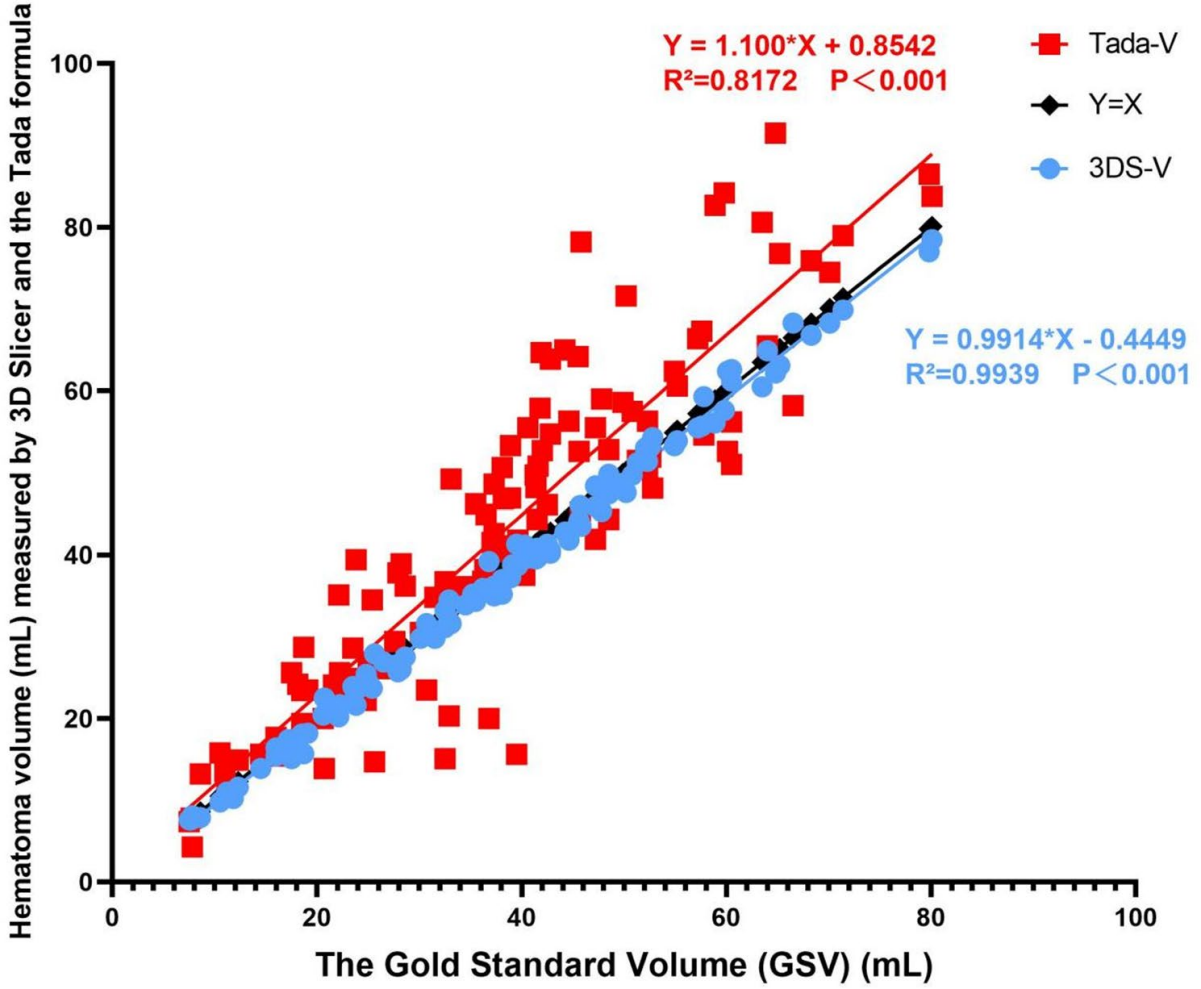
Linear regression analysis comparing measured volumes from 3D Slicer and the Tada formula against the gold standard volume. The blue and red solid lines represent the regression lines for the 3D Slicer and Tada formula, respectively, while the black dashed line denotes the identity line (y = x). A systematic overestimation and proportional bias of the Tada formula are readily apparent

To visually represent the overall relationship between the two measurement methods and the gold standard across the entire volume spectrum, linear regression analysis was performed (Fig. 5).

The regression line for 3D Slicer (blue) nearly coincided with the line of identity (y = x, black dashed line). The regression equation was defined as y = 0.9914x − 0.4449 (R² = 0.9939, *p* < 0.001), statistically confirming its nearly unbiased measurement performance.

In contrast, the regression line for the Tada formula (red) markedly deviated from the line of identity, following the equation y = 1.100x + 0.8542 (R² = 0.8172, *p* < 0.001). A slope greater than unity (1.100) combined with a positive intercept visually confirms the systematic overestimation revealed by Bland-Altman analysis, the magnitude of which increases with larger true hematoma volumes.

### Impact of hematoma morphology and size on volumetric accuracy

The impact of both hematoma morphology and volume on the performance of the two measurement techniques was evaluated through subgroup analyses(Table 3).

#### Stratification by hematoma morphology

For hematomas with regular morphology (*n* = 47, 39.8%), 3D Slicer maintained excellent agreement with the gold standard (bias: -0.35 mL; 95% LoA: -2.20 to 1.50 mL; ICC: 0.998, 95% CI: 0.998–0.999). In the same subgroup, the Tada formula showed substantially higher bias (+ 1.37 mL) with wider limits of agreement (-5.61 to 8.34 mL), despite a relatively high ICC of 0.981 (95% CI: 0.967–0.990).

For irregularly shaped hematomas (*n* = 71, 60.2%), 3D Slicer continued to demonstrate exceptional agreement (bias: -1.05 mL; 95% LoA: -3.87 to 1.78 mL; ICC: 0.996, 95% CI: 0.993–0.997). In contrast, the Tada formula exhibited markedly increased bias (-6.82 mL) with dramatically wider limits of agreement (-27.42 to 13.79 mL) and substantially reduced reliability (ICC: 0.835, 95% CI: 0.748–0.894).

#### Stratification by hematoma volume

As the size of the hematoma increases, the performance of the Tada formula gradually deteriorates. For small hematomas (< 30 mL, *n* = 39, 33.1%), 3D Slicer maintained excellent agreement (bias: -0.64 mL; 95% LoA: -2.75 to 1.48 mL; ICC: 0.985, 95% CI: 0.972–0.992), while the Tada formula already showed notable bias (-2.89 mL) with wide limits of agreement (-12.96 to 7.19 mL) and moderate reliability (ICC: 0.776, 95% CI: 0.613–0.876).

For medium-sized hematomas (30–60 mL, *n* = 66, 55.9%), 3D Slicer continued to perform excellently (bias: -0.86 mL; 95% LoA: -3.40 to 1.67 mL; ICC: 0.986, 95% CI: 0.977–0.991). The Tada formula, however, demonstrated increased bias (-5.73 mL) with exceptionally wide limits of agreement (-25.54 to 14.08 mL) and considerably reduced reliability (ICC: 0.605, 95% CI: 0.426–0.738).

For large hematomas (> 60 mL, *n* = 13, 11.0%), 3D Slicer maintained strong agreement (bias: -0.70 mL; 95% LoA: -4.55 to 3.15 mL; ICC: 0.950, 95% CI: 0.846–0.985). The Tada formula showed persistent bias (-4.41 mL) with very wide limits of agreement (-24.94 to 16.12 mL) and poor reliability (ICC: 0.516, 95% CI: 0.022–0.823).

These results consistently demonstrate that 3D Slicer provides robust and reliable volumetric measurements across all hematoma morphologies and sizes, while the Tada formula exhibits significant measurement errors that are particularly pronounced for irregularly shaped and larger hematomas.

**Table 3:** Comparison of measurement accuracy between the Tada formula and 3D Slicer, stratified by hematoma morphology and size

Volume of hematoma, mL	Number(%)	Method	Bias (vs.GSV)	95% LoA (vs.GSV)	ICC(95% CI) (vs.GSV)
**Groups divided by shape**					
Regular	47(39.8%)	3D Slicer (3DS-V)	-0.35	-2.20-1.50	0.998(0.998–0.999)
		Tada formula (Tada-V)	1.37	-5.61-8.34	0.981(0.967–0.990)
Irregular	71(60.2%)	3D Slicer (3DS-V)	-1.05	-3.87-1.78	0.996(0.993–0.997)
		Tada formula (Tada-V)	-6.82	-27.42-13.79	0.835(0.748–0.894)
**Groups divided by volume**					
< 30 mL	39(33.1%)	3D Slicer (3DS-V)	-0.64	-2.75-1.48	0.985(0.972–0.992)
		Tada formula (Tada-V)	-2.89	-12.96-7.19	0.776(0.613–0.876)
30–60 mL	66(55.9%)	3D Slicer (3DS-V)	-0.86	-3.40-1.67	0.986(0.977–0.991)
		Tada formula (Tada-V)	-5.73	-25.54-14.08	0.605(0.426–0.738)
> 60 mL	13(11.0%)	3D Slicer (3DS-V)	-0.70	-4.55-3.15	0.950(0.846–0.985)
		Tada formula (Tada-V)	-4.41	-24.94-16.12	0.516(0.022–0.823)

## Discussion

The principal finding of this retrospective study demonstrates that the free, open-source 3D Slicer platform achieves exceptional accuracy and reliability for intracerebral hematoma (ICH) volumetry when validated against manual segmentation of high-resolution CTA source images as an authoritative reference standard. Its performance significantly surpasses that of the conventionally used Tada formula, which exhibited substantial and non-negligible systematic overestimation, particularly for irregularly shaped and large hematomas. This finding raises important concerns regarding the continued reliance on the Tada formula for critical therapeutic decision-making, such as assessing surgical intervention thresholds.

Our methodological approach, utilizing CTA source images as the reference standard, effectively addresses the circular reasoning dilemma prevalent in previous literature that validated NCCT-based methods against NCCT itself. However, it is crucial to note that the spatial resolution advantage of CTA in our study was specific to our institutional protocol using submillimeter slices. This may not hold true in centers with different CT acquisition parameters (e.g., thicker slices for NCCT or CTA). Therefore, detailed reporting of imaging parameters should be a standard practice in future studies to allow for proper cross-validation. The sub-millimeter, isotropic properties of CTA source images inherent to our study protocol provide unparalleled spatial resolution, enabling precise discrimination of hematoma boundaries from adjacent vascular structures, thereby establishing a high-level evidence foundation for our conclusions [15]. Furthermore, the high-resolution imaging helped mitigate partial volume effects, which further supports its adoption as the reference standard in this study.

The systematic overestimation of 4.65 mL by the Tada formula represents a clinically significant finding. This inaccuracy originates from its inherent geometrical model deficiency—oversimplifying the complex, often irregular morphology of hematomas into an ideal ellipsoid [16]. Our stratified analysis provides direct evidence: while the Tada formula’s error margin remained relatively acceptable for regular-shaped hematomas, it became substantially amplified for irregular ones [17]. This finding aligns with the pattern observed in prior studies using NCCT as a reference, wherein ABC/2-based formulas exhibit increased error with irregular hematoma morphology. Thus, the novelty of our study lies not in claiming the superiority of 3D Slicer over ABC/2, but in using a more precise reference standard to precisely quantify and confirm the pattern and extent of this error within our cohort. More concerning is the positive correlation between measurement error and hematoma volume, indicating that the Tada formula’s reliability diminishes precisely in cases where measurement accuracy is most critical for guiding treatment decisions. This “less accurate when most needed” characteristic poses a potential risk for clinical decision-making [18]. The clinical relevance is that such systematic overestimation can result in volume misclassification. Patients with Tada-calculated volumes near the surgical threshold could be erroneously triaged to surgery, incurring the risks of an unnecessary invasive intervention. For instance, hematoma expansion is a critical predictor of outcome, and an inaccurate baseline measurement may compromise the assessment of expansion risk and subsequent treatment decisions.

In contrast, 3D Slicer’s voxel-based computational approach entirely bypasses geometrical assumptions, achieving near-perfect accuracy. Our results are in line with previous studies that applied 3D Slicer in other contexts, such as brain tumor segmentation, further supporting its robustness for achieving precise volumetric measurements [19, 20]. Although its semi-automated segmentation process requires more time (approximately 5–10 min per case) compared to the Tada formula (< 1 min), the substantial gain in precision justifies this investment, particularly for clinical trials, prognostic modeling, and precision surgical planning where measurement accuracy is paramount. We strongly recommend adopting 3D Slicer-based volumetric analysis as the standard measurement method for designing prospective clinical trials and formulating individualized surgical strategies. For routine emergency assessments, clinicians must maintain awareness of the Tada formula’s limitations—when measurements approach critical decision thresholds, significant overestimation should be suspected. The free and open-source nature of 3D Slicer eliminates economic barriers to its global implementation, including resource-limited healthcare settings, holding considerable potential to transform clinical practice paradigms.

Several limitations warrant acknowledgment. First, the retrospective design inherently carries risk of selection bias, though we mitigated this through consecutive case enrollment. Second, while CTA manual segmentation represents the current optimal in vivo standard, it cannot be considered an indisputable “gold standard” (such as pathological volume measurement). Although employing manual CTA segmentation as a reference was a practical necessity for this comparison, this approach risks conflating contrast extravasation (“spot sign”) with the hematoma and is insensitive to acute expansion [21]. Thus, it is not an absolute gold standard for volume assessment. Future studies could explore the role of multimodal image fusion, for instance, to better differentiate contrast extravasation from acute hematoma or to improve the characterization of hemorrhage complexity. Finally, although 3D Slicer’s efficiency surpasses fully manual segmentation, it remains inferior to the Tada formula. Future work should focus on developing and integrating artificial intelligence-based fully automatic segmentation algorithms that maintain 3D Slicer’s accuracy while dramatically improving workflow efficiency, ultimately bridging the transition from research tool to routine clinical application.

## Conclusion

This study demonstrates that validation against high-resolution CTA source images confirms 3D Slicer as a reliable and superior tool for accurate volumetric quantification of intracerebral hemorrhage. The Tada formula exhibits inherent systematic bias and only moderate agreement with the reference standard, which substantially limits its utility in the context of modern precision medicine. The adoption of 3D Slicer-based quantitative analysis is therefore recommended in both academic research and high-quality clinical practice to enhance the objectivity and scientific rigor of ICH management and scientific investigation.

## Data Availability

The data supporting the findings of this study are available upon reasonable request from the corresponding author.

## References

[1] Magid-Bernstein J, Girard R, Polster S, et al. Cerebral hemorrhage: Pathophysiology, Treatment, and future directions. Circ Res. 2022;130(8):1204–29. 35420918 10.1161/CIRCRESAHA.121.319949 PMC10032582

[2] Hostettler IC, Seiffge DJ, Werring DJ. Intracerebral hemorrhage: an update on diagnosis and treatment. Expert Rev Neurother. 2019;19(7):679–94. 31188036 10.1080/14737175.2019.1623671

[3] LoPresti MA, Bruce SS, Camacho E, et al. Hematoma volume as the major determinant of outcomes after intracerebral hemorrhage. J Neurol Sci. 2014;345(1–2):3–7. 25034055 10.1016/j.jns.2014.06.057

[4] Xu X, Chen X, Zhang J, et al. Comparison of the Tada formula with software slicer: precise and low-cost method for volume assessment of intracerebral hematoma. Stroke. 2014;45(11):3433–5. 25316277 10.1161/STROKEAHA.114.007095

[5] Webb AJS, Ullman NL, Morgan TC, et al. Accuracy of the ABC/2 score for intracerebral hemorrhage: systematic review and analysis of MISTIE, CLEAR-IVH, and CLEAR III. Stroke. 2015;46(9):2470–6. 26243227 10.1161/STROKEAHA.114.007343 PMC4550520

[6] Huttner HB, Steiner T, Hartmann M, et al. Comparison of ABC/2 Estimation technique to computer-assisted planimetric analysis in warfarin-related intracerebral parenchymal hemorrhage. Stroke. 2006;37(2):404–8. 16373654 10.1161/01.STR.0000198806.67472.5c

[7] Oge DD, Arsava EM, Pektezel MY, et al. Intracerebral hemorrhage volume estimation: is modification of the ABC/2 formula necessary according to the hematoma shape? Clin Neurol Neurosurg. 2021;207:106779. 34214866 10.1016/j.clineuro.2021.106779

[8] Zhao B, Jia W-B, Zhang L-Y, et al. 1/2SH: A Simple, Accurate, and reliable method of calculating the hematoma volume of spontaneous intracerebral hemorrhage. Stroke. 2020;51(1):193–201. 31795899 10.1161/STROKEAHA.119.026951

[9] Greenberg SM, Ziai WC, Cordonnier C, et al. 2022 guideline for the management of patients with spontaneous intracerebral hemorrhage: A guideline from the American heart association/American stroke association. Stroke. 2022;53(7):e282–361. 35579034 10.1161/STR.0000000000000407

[10] Sha Z, Zhang X, Li Z, et al. Improvements of the Tada formula in estimating the intracerebral hemorrhage volume based on computed tomography. Quant Imaging Med Surg. 2023;13(7):4268–83. 37456319 10.21037/qims-22-1084 PMC10347354

[11] Fedorov A, Beichel R, Kalpathy-Cramer J, et al. 3D slicer as an image computing platform for the quantitative imaging network. Magn Reson Imaging. 2012;30(9):1323–41. 22770690 10.1016/j.mri.2012.05.001 PMC3466397

[12] You Y, Niu Y, Sun F, et al. Three-dimensional printing and 3D slicer powerful tools in Understanding and treating neurosurgical diseases. Front Surg. 2022;9:1030081. 36311943 10.3389/fsurg.2022.1030081 PMC9614074

[13] Wannamaker R, Buck B, Butcher K. Multimodal CT in acute stroke. Curr Neurol Neurosci Rep. 2019;19(9):63. 31352585 10.1007/s11910-019-0978-z

[14] Sallustio F, Motta C, Pizzuto S, et al. CT angiography ASPECTS predicts outcome much better than Noncontrast CT in patients with stroke treated endovascularly. AJNR Am J Neuroradiol. 2017;38(8):1569–73. 28619833 10.3174/ajnr.A5264 PMC7960404

[15] Xu R, Zhao D, Wen R, et al. Regional reliability of combining CT Angiography-source image and Non-contrast CT in acute ischemic stroke. Int J Med Sci. 2024;21(13):2623–9. 39439465 10.7150/ijms.101166 PMC11492883

[16] Maeda AK, Aguiar LR, Martins C, et al. Hematoma volumes of spontaneous intracerebral hemorrhage: the ellipse (ABC/2) method yielded volumes smaller than those measured using the planimetric method. Arq Neuropsiquiatr. 2013;71(8):540–4. 23982013 10.1590/0004-282X20130084

[17] Delcourt C, Carcel C, Zheng D, et al. Comparison of ABC methods with computerized estimates of intracerebral hemorrhage volume: the INTERACT2 study. Cerebrovasc Dis Extra. 2019;9(3):148–54. 31838472 10.1159/000504531 PMC6940457

[18] Boulouis G, van Etten ES, Charidimou A, et al. Association of key magnetic resonance imaging markers of cerebral small vessel disease with hematoma volume and expansion in patients with Lobar and deep intracerebral hemorrhage. JAMA Neurol. 2016;73(12):1440–7. 27723863 10.1001/jamaneurol.2016.2619 PMC5584595

[19] Hernandez-Duran S, Walter J, Behmanesh B, et al. Surgical infarct volume reduction and functional outcomes in patients with ischemic cerebellar stroke: results from a multicentric retrospective study. J Neurosurg. 2024;141(6):1681–6. 38941630 10.3171/2024.3.JNS232883

[20] Khadhraoui E, Schmidt L, Klebingat S, et al. Comparison of a new MR rapid wash-out map with MR perfusion in brain tumors. BMC Cancer. 2024;24(1):1139. 39267002 10.1186/s12885-024-12909-z PMC11395865

[21] Ederies A, Demchuk A, Chia T, et al. Postcontrast CT extravasation is associated with hematoma expansion in CTA spot negative patients. Stroke. 2009;40(5):1672–6. 19286577 10.1161/STROKEAHA.108.541201

